# Sarcopenia, frailty, and elective surgery outcomes in the elderly: an observational study with 125 patients (the SAFESOE study)

**DOI:** 10.3389/fmed.2023.1185016

**Published:** 2023-08-07

**Authors:** Isabel Chateaubriand Diniz de Salles, Renato Sernik, José Luiz Padilha da Silva, Cesar Taconeli, Ana Alice Amaral, Christina May Moran de Brito, Ana Luiza Bierrenbach

**Affiliations:** ^1^Hospital Sírio Libanês, Instituto de Ensino e Pesquisa, São Paulo, Brazil; ^2^Departamento de Estatística, Universidade Federal do Paraná, Curitiba, Brazil

**Keywords:** sarcopenia, frailty, ultrasonography, aged, postoperative complications

## Abstract

**Background:**

Sarcopenia is a syndrome characterized by loss of muscle mass, strength and function. Frailty, a state of vulnerability with diminished reserves. The measurement of perioperative risk does not include the assessment of these variables, as little is known about how these conditions impact each other.

**Methods:**

Observational study with a cross-sectional and a prospective cohort component. Elderly people over 60 years of age, able to walk and to independently perform activities of daily living were consecutively recruited in the preoperative period of non-emergency surgical procedures. Frailty was measured by the modified frailty index (mFI-11). Sarcopenia was measured by: (1) thickness and echogenicity on ultrasound; (2) handgrip strength on dynamometry and (3) gait speed. Data obtained from eight muscle groups were submitted to Principal Component Analysis. Postoperative complications were measured using the Clavien-Dindo scale. Follow-up was performed for 1 year to record readmissions and deaths.

**Results:**

Between February and May 2019, 125 elderly people were recruited, median age of 71 years (IQR 65–77), 12% of whom were frail. Frailty was associated with older age, use of multiple medicines, presence of multimorbidity and greater surgical risk according to the American Society of Anesthesiologists (ASA) scale, in addition to lower gait speeds and lower handgrip strength. Frailty was also independently associated with smaller measurements of muscle thickness but not with echogenicity, and with longer hospital and Intensive care unit (ICU) stays. Prevalence of sarcopenia was 14% when considering at least two criteria: low walking speed and low handgrip strength. For muscle thickness, lower values were associated with female gender, older age, frailty, lower gait speeds and lower muscle strength, higher proportion of postoperative complications and higher occurrence of death. For echogenicity, higher values were related to the same factors as those of lower muscle thickness, except for postoperative complications. Lower gait speeds and lower handgrip strength were both associated with higher proportions of postoperative complications, and longer hospital stays. A higher mortality rate was observed in those with lower gait speeds.

**Conclusion:**

Sarcopenia was associated with frailty in all its domains. Unfavorable surgical outcomes were also associated with these two conditions.

## 1. Introduction

Sarcopenia is a progressive and generalized muscle disease related to aging, characterized by loss of muscle mass, quality, and strength, with an impact on function, resulting in some degree of physical disability, loss of quality of life and increased mortality (1, 2). The prevalence and impact of sarcopenia vary substantially, as they depend on the definition applied, staging, and choice of instruments used for measurement, as well as the age range of the considered population (3). Sarcopenia is believed to be the main risk factor for aging-related functional decline (4, 5). Sarcopenia in the elderly and in patients with several comorbidities is related to prolonged hospitalizations, with higher rates of complications, especially infections, and higher overall mortality (6, 7). Impaired muscle function is an independent predictor of hospitalization, disability, and death (8).

Frailty is a state of vulnerability manifested by diminished physiological reserves that affect the ability to maintain homeostasis in the event of exposure to stressors, resulting in increased risk for adverse health outcomes (9–15). For a fragile patient, stressors can result in serious consequences, such as increased dependence on caregivers and a greater predisposition to falls and delirium. Frailty prevalence rates also vary widely, and this discrepancy can be explained by the lack of consensus on the definition applied, different instruments used for assessment, demographic characteristics, and particularities of the studied population (16, 17).

Elderly patients are at increased risk of postoperative complications (18), which require rapid recognition and treatment, particularly in this population, otherwise they can frequently lead to a cascade of events that may result in loss of independence and worsening of quality of life, higher treatment-related costs, some degree of disability, and greater mortality (19). Studies suggest that frailty is a condition that affects a large proportion of elderly patients who will undergo surgery (20–22).

In this scenario, the present study aimed to evaluate the association between sarcopenia, in its three domains, with frailty, in a population of elderly people admitted to hospital to undergo non-emergency surgical procedures. The association of sarcopenia with frailty is still unclear in the literature. There are studies that show the influence of sarcopenia on frailty overtime (23). Moreover, as a secondary objective, to evaluate the correlation of sarcopenia and frailty with postsurgical outcomes, as sarcopenia and frailty seem to have significant adverse impacts on the occurrence of postoperative outcomes (24).

## 2. Methods

An observational study was conducted in the preoperative units of the Hospital Sírio-Libanês, in São Paulo, Brazil–a tertiary, philanthropic institution with a 500-bed capacity. The participants were elderly people over 60 years of age, able to walk, with or without walking aids, admitted to hospital for non-emergency surgical procedures, who signed the Free and Informed Consent Term. Those with neuromuscular diseases, paresis, paralysis or limb amputation, dementia, and that were dependent for daily life activities, or lived with homecare, or were institutionalized were excluded. Patients were recruited consecutively, without prior selection. The initial interviews and measurements took place on the eve of the procedure and lasted an average of 20 min.

In the preoperative period, data were collected to characterize the population (Appendix 1 in Supplementary material). Frailty was characterized by the Modified Frailty Index 11 (which considers the presence of comorbidities and the functional status during the 30 days before surgery). Each one of the present comorbidities add one point, frailty adds on 3 or more points, and pre-frailty puts on 1 or 2 points (16, 17). The assessment of muscle mass was characterized using ultrasound measurement of the cross-sectional area of eight muscle groups: biceps brachii and brachii; rectus femoris; vastus intermedius; rectus abdominis; external and internal obliques; transversus abdominis; and medial gastrocnemius (Appendix 2 in Supplementary material). The percentage of fat was assessed by bioimpedance test (25). Muscle strength was measured with the use of a handgrip dynamometer (8, 9). Muscle function assessed by the 15-meter walk test (16, 17, 25, 26).

In the postoperative period, data related to surgical complications were collected daily, using the Clavien-Dindo Classification (Appendix 3 in Supplementary material), from the day after surgery until discharge (27). Hospital mortality and total length of stay were also computed. Mortality after discharge and hospital readmissions were accessed through telephone contact with the family or guardians, on the first, third and twelfth month after discharge.

Regarding the statistical analysis, categorical data were summarized by their absolute and relative frequencies and compared with the use of the Pearson’s Chi-square or Fisher’s Exact Test. Continuous data were summarized by their mean and standard deviations or by median and inter-quartile percentiles. When the data had a normal distribution, the Student’s t-test was used to compare two independent groups and the Analysis of Variance (ANOVA) and the F-test were used to compare three or more groups. When data were not normally distributed, the Wilcoxon-Mann–Whitney test was used when comparing two independent groups and the Kruskal-Wallis test was used when comparing three or more groups.

For the analysis of data on the thickness and echogenicity of muscle groups, we used the Principal Component Analysis (PCA). PCA was used to explore the correlation structure between the thickness and echogenicity measurements. In addition, it allowed us to reduce the dimension of the problem, by producing two new variables (principal components) able to explain a substantial amount of the original variation, registered by the eight thickness (or echogenicity). Parallel analysis was used to decide the number of principal components to be retained and studied. In the parallel analysis, new datasets are simulated completely at random, and the corresponding components are obtained. Then, the original components are compared with the simulated ones, and only those explaining more than the simulated ones should be considered. The principal components are defined by interpretable linear combinations based on the original variables, where the coefficients of each original variable are related to the correlations between the original variables and the components. Principal component analysis is a usual multivariate statistical method, largely used in medical sciences (28, 29).

The Hypothesis Tests of the association of the scores of the components 1 and 2 were performed separately with the main variables of the study, with the use of the tests for means described above. The Spearman’s correlation coefficient was calculated for the continuous variable “length of stay.” Interactions were also evaluated. Conclusions were based on a 5% significance level. All analysis were performed using the R statistical software, version 4.0.292. The R package psych was used to perform the principal component and parallel analysis.

## 3. Results

Between February and May 2019, 125 elderly people were recruited during the preoperative period of non-emergency surgeries. The studied group of patients had a slight male predominance (52.8%) and an average age of 71 years (IQR 65–77). The patients used a median of 4 different medications (IQR 2–6), had an average of 3 comorbidities (IQR 2–4), and an ASA score distributed as follows: 12 (9.6%) at level 1; 85 (68%) at level 2; and 28 (22.4%) at level 3. Regarding frailty, 77 (61.6%) were not frail, 33 (26.4%) were pre-frail, and 15 (12%) were frail. It was observed that older people, those who took more medications, had more comorbidities, and had higher ASA scores were more often considered frail (value of ps ≤0.001).

Of the 125 patients, 34 (28.3%) had low gait speed (the cut-off points for gait speed was ≤0.8 m/s). There were 20 (16%) patients with handgrip strength below the expected limit (the cut-off points for dynamometry were ≤ 27 kgf for men and ≤ 16 kgf for women). There were 38 (30.4%) patients with gait speed below 0.8 m/s or handgrip strength below the expected limit. Sarcopenia, measured by the 15-meter gait speed test and by the handgrip strength dynamometry, was positively correlated with frailty (Table 1).

**Table 1:** Distribution of measurements of gait speed and strength, by degrees of frailty.

Categories*	Normal (*N* = 77)	Pre-frail (*N* = 33)	Frail (*N* = 15)	Total (*N* = 125)	*p*-value**
*Gait speed (m/s)*					
	1.1 (0.9–1.2)	0.9 (0.6–1.1)	0.6 (0,3–0.8)	1.0 (0.8–1.2)	<0.001
*Gait speed – cut-off point of 0.8 m/s*					
<=0.8 m/s	13 (17.6)	10 (32.3)	11 (73.3)	34 (28.3)	<0.001
>0.8 m/s	61 (82.4)	21 (67.7)	4 (26.7)	86 (71.7)	
*Dynamometer – handgrip strength – different cut-off points by sex* ***					
Normal	72 (93.5)	26 (78.8)	7 (46.7)	105 (84)	<0.001
Below the cut-offs	5 (6.5)	7 (21.2)	8 (53.3)	20 (16)	

* Data are presented as median (IQR) for continuous measures and n (%) for categorical measures.

** Kruskal–Wallis test for continuous measurements and Fisher’s exact or chi-square test for categorical measurements.

*** Cut-off points for dynamometry: < or > =27 kgf for men and < or > =16 kgf for women.

The other parameters used to characterize sarcopenia, which were muscle thickness and echogenicity obtained by ultrasound, are presented in Table 2, separated into the degrees of frailty. Lower values of muscle thickness were associated with frailty, except for the biceps and brachii, medial gastrocnemius, and rectus abdominis. Nevertheless, for echogenicity, there was no relationship between this measurement and frailty.

**Table 2:** Distribution of measurements of each muscle thickness and echogenicity, by degrees of frailty.

Muscles*	Normal (*N* = 77)	Pre-frail (*N* = 33)	Frail (*N* = 15)	Total (*N* = 125)	*p*-value**
*Thickness (cm)* *					
Biceps brachii and Brachialis	4.0 (3.7–4.5)	4.1 (3.7–4.6)	3.5 (3.2–4.4)	4.0 (3.6–4.5)	0.15
Rectus femoris	1.2 (1.0–1.5)	1.2 (1.0–1.5)	0.9 (0.8–1.3)	1.2 (1.0–1.5)	0.020
Vastus intermediate	1.1 (0.9–1.4)	1.2 (0.9–1.5)	0.8 (0.7–1.1)	1.1 (0.9–1.4)	0.002
Quadriceps femoris	2.3 (2.0–2.8)	2.5 (2.0–2.9)	1.7 (1.5–2.2)	2.3 (1.9–2.8)	0.005
Rectus abdominis	0.8 (0.7–1.0)	0.9 (0.7–1.0)	0.9 (0.7–0.9)	0.8 (0.7–1.0)	0.81
External oblique	0.5 (0.4–0.6)	0.5 (0.4–0.6)	0.4 (0.3–0.5)	0.5 (0.4–0.6)	0.002
Internal oblique	0.5 (0.4–0.7)	0.5 (0.4–0.8)	0.4 (0.3–0.5)	0.5 (0.4–0.7)	0.004
Transversus abdominis	0.4 (0.3–0.4)	0.4 (0.2–0.4)	0.2 (0.2–0.3)	0.3 (0.3–0.4)	<0.001
Medial gastrocnemius	1.5 (1.3–1.7)	1.6 (1.3–1.7)	1.3 (1.1–1.5)	1.5 (1.3–1.7)	0.060
*Echogenicity (grayscale)* *					
Biceps brachii and Brachialis	60.5 (51.8–68.1)	62.9 (53.5–68.5)	64.2 (58.6–72.7)	61.8 (52.8–68.5)	0.35
Rectus femoris	52.9 (44.1–62.5)	54.4 (45.1–63.5)	55.9 (50.4–73.1)	53.5 (45.1–63.3)	0.57
Vastus intermediate	49.9 (41.0–61.8)	47.3 (36.7–62.8)	64.7 (50.2–79.1)	50.8 (38.7–63.9)	0.092
Quadriceps femoris	57.7 (51.6–67.3)	59.4 (53.3–68.4)	65.1 (56.6–76.3)	59.4 (52.1–68.2)	0.19
Rectus abdominis	61.9 (51.1–73.9)	64.5 (55.8–76.7)	61.5 (47.0–71.9)	63.3 (52.5–74.3)	0.48
External oblique	70.3 (59.2–80.9)	71.7 (62.9–79.2)	69.4 (66.8–76.2)	69.9 (62.5–80.0)	0.92
Internal oblique	51.0 (43.0–64.1)	50.9 (47.9–59.3)	57.2 (46.8–64.7)	52.0 (44.9–64.1)	0.57
Transversus abdominis	46.9 (38.6–53.0)	47.4 (35.5–57.1)	56.1 (42.7–60.5)	47.8 (38.7–56.1)	0.081
Medial gastrocnemius	46.3 (40.4–52.4)	48.4 (35.4–54.8)	52.5 (44.7–66.7)	47.0 (40.4–55.3)	0.17

* Data are presented as median (IQR).

** Kruskal–Wallis tests.

In Table 3, we describe the relationship between frailty and unfavorable outcomes. There was a significant correlation between frailty and length of stay, and, also, postoperative complications. The most frequent types of complications, considering the Clavien-Dindo Classification, were grades I (20.8%) and II (11.2%). There was no correlation between frailty and readmissions or death, considering the one-year follow-up period.

**Table 3:** Distribution of postoperative complications, length of stay, readmissions, and death up to 1 year of follow-up, separated by degrees of frailty.

Categories*	Normal (*N* = 77)	Pre-frail (*N* = 33)	Frail (*N* = 15)	Total (*N* = 125)	*p*-value**
*Postoperative complications – Clavien-Dindo scale*					
No complications	51 (66.2%)	19 (57.7%)	5 (33.3%)	75 (60.0%)	0.049
I	13 (16.9%)	10 (30.3%)	3 (20.0%)	26 (20.8%)	
II	7 (9.1%)	1 (3.0%)	6 (40.0%)	14 (11.2%)	
IIIa	1 (1.3%)	1 (3.0%)	1 (6.7%)	3 (2.4%)	
IIIb	1 (1.3%)	0 (0.0%)	0 (0.0%)	1 (0.8%)	
Iva	2 (2.6%)	1 (3.0%)	0 (0.0%)	3 (2.4%)	
IVb	1 (1.3%)	0 (0.0%)	0 (0.0%)	1 (0.8%)	
V	1 (1.3%)	1 (3.0%)	0 (0.0%)	2 (1.6%)	
*Length of stay (days)*					
	2.0 (1.0–3.0)	2.0 (1.0–4.0)	4.0 (2.0–15.0)	2.0 (1.0–4.0)	0.037
*Readmission within 1 year of hospital discharge*					
No	50 (64.9%)	25 (75.8%)	10 (66.7%)	85 (68.0%)	0.392***
Yes	24 (31.2%)	7 (21.2%)	3 (20.0%)	34 (27.2%)	
Not informed	3 (3.9%)	1 (3.0%)	2 (13.3%)	6 (4.8%)	
*Death*					
No	74 (96.1%)	32 (97.0%)	14 (93.3%)	120 (96.0%)	0.645
Yes	3 (3.9%)	1 (3.0%)	1 (6.7%)	5 (4.0%)	

* Data are presented as median (IIR) for continuous measures and n (%) for categorical measures.

** Kruskal–Wallis test for continuous measurements and Fisher’s Exact or Chi-square tests for categorical measurements.

*** Excluding the “not informed” category, the p value was equal to 0.516.

Table 4 shows that lower gait speeds were associated with higher proportions of postoperative complications, longer length of stay, and higher occurrence of death. Similarly, Table 5 shows that lower handgrip strengths were associated with higher proportions of postoperative complications and longer length of stay, but there were no associations with readmission and death rates.

**Table 4:** Distribution of the studied outcomes: length of stay (days), readmission, and death due to any cause in up to 1 year of follow-up, separated into two categories of gait speed.

Categories*	Gait speed			*p*-value**
	>0.8 m/s (*N* = 86)	<=0.8 m/s (*N* = 34)	Total (*N* = 120)	
Postoperative complications – binary variable				
No	59 (68.6%)	16 (47.1%)	75 (62.5%)	0.037
Yes	27 (31.4%)	18 (52.9%)	45 (37.5%)	
Length of stay (days)				
	2.0 (1.0–3.0)	4.0 (2.0–10.0)	2.0 (1.0–4.0)	<0.001
Readmission within 1 year of hospital discharge				
No	61 (70.9%)	21 (61.8%)	82 (68.3%)	0.605***
Yes	22 (25.6%)	11 (32.3%)	33 (27.5%)	
Not informed	3 (3.5%)	2 (5.9%)	5 (4.2%)	
Death				
No	85 (98.8%)	30 (88.2%)	115 (95.8%)	0.022
Yes	1 (1.2%)	4 (11.8%)	5 (4.2%)	

* Data are presented as median (IIR) for continuous measures and *n* (%) for categorical measures.

** Kruskal-Wallis test for continuous measurements and Fisher’s Exact or Chi-square tests for categorical measurements.

*** Excluding the “not informed” category, the *p* value was equal to 0.705.

**Table 5:** Distribution of outcomes studied: length of stay (days), readmissions, and death due to any cause within up to 1 year of follow-up, separated into two categories of handgrip strength.

Categories*	Handgrip strength (dynamometer)			*p*-value**
	Normal (*n* = 105)	Below the cut-offs (*N* = 25)	Total (*N* = 120)	
Postoperative complications – binary variable				
No	68 (64.8%)	7 (35.0%)	75 (60.0%)	0.023
Yes	37 (35.2%)	13 (65.0%)	50 (40.0%)	
Length of stay (days)				
	0.0 (0.0–0.0)	0.5 (0.0–2.0)	0.0 (0.0–0.0)	<0.001
Readmission within 1 year of hospital discharge				
No	71 (67.6%)	14 (70.0%)	85 (68.0%)	0.331***
Yes	30 (28.6%)	4 (20.0%)	34 (27.2%)	
Not informed	4 (3.8%)	2 (10.0%)	6 (4.8%)	
Death				
No	102 (97.1%)	18 (90.0%)	120 (96.0%)	0.181
Yes	3 (2.9%)	2 (10.0%)	5 (4.0%)	

* Data are presented as median (IIR) for continuous measures and *n* (%) for categorical measures.

** Kruskal-Wallis test for continuous measurements and Fisher’s Exact or Chi-square tests for categorical measurements.

*** Excluding the “not informed” category, the *p*-value was equal to 0.811.

**Figure 1:**
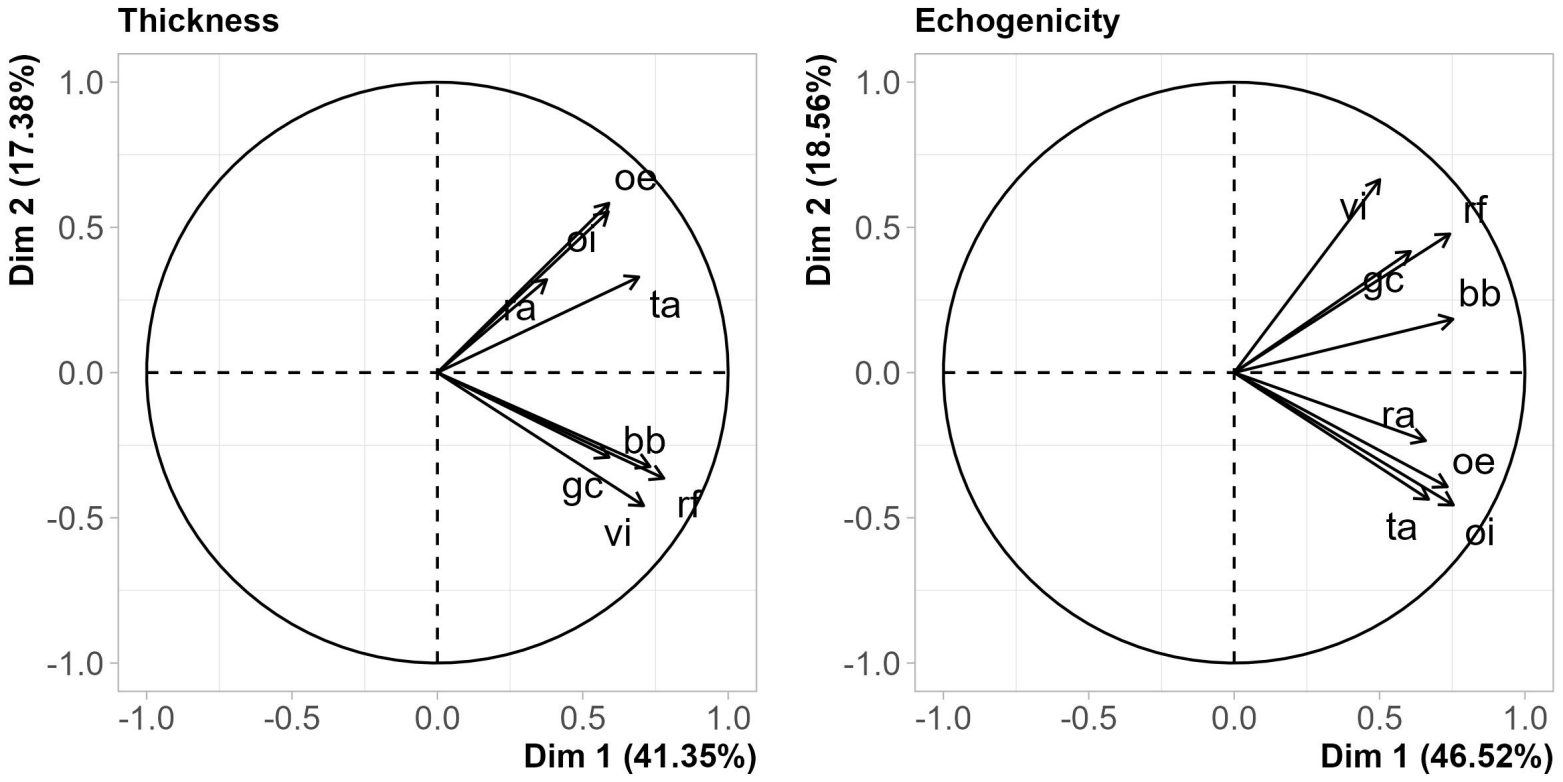
Principal component analysis of thickness and echogenicity measurements. The horizontal axis stands for the first principal component, the vertical, the second, whereas the arrows represents the original variables. Arrows with similar orientations show positively correlated variables.

For the summary of the analysis of the eight muscle groups of the entire studied population, we used the statistical method Principal Component Analysis. Two principal components (or dimensions) were identified. The first principal component (PC1) assigned positive loadings for all thickness and echogenicity variables, i.e., all muscles were positively correlated with each other, that is, in an individual, if a muscle tended to be thinner or more echogenic, such characteristics tended to be replicated in other muscle groups. In other words, there is a congruence of measurements across the eight muscle groups. Vectors in Figure 1 (thickness and echogenicity) are all pointing toward the same direction in relation to the horizontal axis. The second principal component (PC2), on the other hand, assigned loadings with different sets of variables, and it can be interpreted as a contrast or divergence between the muscular behavior for appendicular and abdominal muscles. Component 2 is represented by the fact that the arrows for the appendicular muscles and those of the abdominal muscles are pointing toward opposite direction in relation to the vertical axis. The two principal components jointly explain 58.728 and 65.084% of the original variance for the thickness and echogenicity variables, respectively.

The remaining results show the association of the principal components with the other study variables. For muscle thickness, higher values of component 1 were associated with male gender, younger age, lower degrees of frailty, higher gait speeds and normal handgrip strength measurements by dynamometry, absence of postoperative complications of grade II or higher on the Clavien-Dindo Classification, and not dying. There was no association of the component 1 with readmission for up to 1 year. There was a correlation of the component 2 with gender, that is, the thickness divergence between the appendicular and abdominal muscles occurred differently between the sexes: for men, the thickness of the appendicular muscles is greater than those presented by women. There were no other associations of the component 2 with other variables (Table 6).

**Table 6:** Association between the principal components that summarize the thickness and echogenicity measurements and other categorical variables.

Variable	Category	*N* (%)	Comp. 1*	*p*-value (1)**	Comp. 2*	*p*-value (2)**
*Thickness*						
Sex	Male	66 (52.8)	0.648 (1.777)	<0.001	−0.444 (1.199)	<0.001
	Female	59 (47.2)	−0.725 (1.605)		0.497 (0.953)	
Age	(60–69)	58 (46.4)	0.697 (1.713)	<0.001	0.102 (1.217)	0.666
	(70–79)	46 (36.8)	−0.250 (1.701)		−0.076 (1.189)	
	(80+)	21 (16.8)	−1.377 (1.502)		−0.117 (1.109)	
Frailty binary	Normal	110 (88.0)	0.240 (1.704)	0.001	0.035 (1.195)	0.354
	Frail	15 (12.0)	−1.761 (1.774)		−0.255 (1.098)	
Frailty categories	Normal	77 (61.6)	0.190 (1.587)	<0.001	0.069 (1.217)	0.608
	Pre-frail	33 (26.4)	0.358 (1.973)		−0.045 (1.159)	
	Frail	15 (12.0)	−1.761 (1.774)		−0.255 (1.098)	
Gait speed	>0.8 m/s	86 (71.7)	0.541 (1.649)	<0.001	−0.032 (1.206)	0.713
	<=0.8 m/s	34 (28.3)	−1.429 (1.572)		0.055 (1.149)	
Dynamometry	Normal	105 (84.0)	0.385 (1.615)	<0.001	0.008 (1.226)	0.848
	Weak	20 (16.0)	−2.023 (1.536)		−0.040 (0.957)	
Postoperative complications	No	75 (60.0)	0.187 (1.702)	0.176	−0.061 (1.270)	0.464
	Yes	50 (40.0)	−0.280 (1.982)		0.092 (1.047)	
Readmission	No	85 (71.4)	−0.062 (1.784)	0.560	0.084 (1.139)	0.483
	Yes	34 (28.6)	0.157 (1.858)		−0.089 (1.233)	
Death	No	120 (96.0)	0.087 (1.796)	0.019	0.012 (1.204)	0.254
	Yes	5 (4.0)	−2.080 (1.328)		−0.283 (0.465)	
*Echogenicity*						
Sex	Male	66 (52.8)	−0.669 (1.991)	<0.001	−0.288 (1.153)	0.005
	Female	59 (47.2)	0.748 (1.581)		0.322 (1.229)	
Age	(60–69)	58 (46.4)	−0.481 (1.921)	0.032	−0.142 (1.169)	0.287
	(70–79)	46 (36.8)	0.469 (1.863)		0.020 (1.186)	
	(80+)	21 (16.8)	0.302 (1.914)		0.350 (1.427)	
Frailty binary	Normal	110 (88.0)	−0.102 (1.994)	0.035	−0.038 (1.167)	0.474
	Frail	15 (12.0)	0.745 (1.266)		0.276 (1.598)	
Frailty categories	Normal	77 (61.6)	−0.162 (1.964)	0.252	−0.018 (1.145)	0.631
	Pre-frail	33 (26.4)	0.040 (2.085)		−0.084 (1.236)	
	Frail	15 (12.0)	0.745 (1.266)		0.276 (1.598)	
Gait speed	>0.8 m/s	86 (71.7)	−0.489 (1.984)	<0.001	−0.166 (1.170)	0.018
	<=0.8 m/s	34 (28.3)	1.120 (1.279)		0.445 (1.257)	
Dynamometry	Normal	105 (84.0)	−0.245 (1.912)	<0.001	−0.123 (1.162)	0.026
	Weak	20 (16.0)	1.286 (1.551)		0.646 (1.360)	
Postoperative complications	No	75 (60.0)	−0.091 (1.998)	0.516	−0.134 (1.219)	0.133
	Yes	50 (40.0)	0.137 (1.854)		0.202 (1.215)	
Readmission	No	85 (71.4)	0.071 (1.977)	0.384	0.027 (1.192)	0.600
	Yes	34 (28.6)	−0.271 (1.895)		−0.098 (1.149)	
Death	No	120 (96.0)	−0.034 (1.968)	0.010	−0.035 (1.230)	0.048
	Yes	5 (4.0)	0.827 (0.477)		0.832 (0.703)	

* Mean (standard deviation) for the scores of the respective principal components.

** Mean comparison tests (*t* test for two groups and *F* test for three groups).

For echogenicity, higher values of component 1 were associated with female gender, older age, higher degrees of frailty (although only frailty categorized as a binary variable had a value of *p* <5%), lower gait speeds, lower handgrip strength measurements by dynamometry, and death (Table 6). There was an association of the component 2 with female gender, lower gait speeds and lower handgrip strength measurements by dynamometry, in addition to death - meaning that the echogenicity of the appendicular muscles, relative to those of the axial muscles, was higher for women, for people with low walking speed gait and low handgrip strength by dynamometry (Table 6).

## 4. Discussion

This study aimed to establish the association between frailty and sarcopenia in a series of 125 elderly subjects in the preoperative period of non-emergency surgery. As a secondary objective, we also sought to correlate sarcopenia and frailty with unfavorable outcomes, such as: longer hospital stays, readmissions, death, and postoperative complications.

Considering frailty, the prevalence found in our study was 12%, similar to other studies, which indicate the general prevalence of frailty from 10.7 to 13.6%. Interestingly enough, the prevalence of frailty in the elderly population varies from 4.0 to 59.1%, depending on the definition used and the population studied (16). The scale chosen to assess frailty in our study was the Modified Frailty Index 11 (mF11), which is already well established in the literature, and represents an accurate predictor of mortality and postoperative complications in surgery (30).

In our study, frailty was related to older age, but not to gender. This finding is coincident with the literature. Criteria for frailty were found in 16.7% of males and 6.8% of females (Appendix 1 in Supplementary material), differing from authors such as Collard and Fried, who found that the prevalence of frailty increases with age but is higher in women (16, 17, 31).

The prevalence of sarcopenia was 30.4% (Table 1), with no significant gender differences, when considering at least one criterion: gait speed below 0.8 m/s or handgrip strength below the expected limit for sex group. When considering at least two criteria, as recommended by the European consensus, the prevalence of sarcopenia in our study approaches the value of 14%, similar to that observed in the literature (32). The prevalence of sarcopenia varies enormously depending on the definition and instrument used to define it and the population studied. In studies carried out in the general population, the prevalence of sarcopenia in adults aged 60 to 70 years varies between 5 and 13%, increasing to 11 to 50% in those aged 80 years and over (33). In a meta-analysis that pooled studies with the definition of sarcopenia based on the European Working Group on Sarcopenia in Older People (EWGSOP) 2019 consensus, in healthy patients, aged 60 years and over, the overall estimates were 3.96% (32). In our study, the prevalence of sarcopenia, as defined by the European consensus, was slightly higher than that of frailty. It is important to keep in mind that frailty constitutes a broader functional spectrum compared to sarcopenia (34).

Frailty was correlated with sarcopenia, both in terms of loss of muscle strength and low gait speed, both conditions predefined by the EWGSOP consensus (32). Regarding the muscle mass criterion, frailty was correlated with smaller thicknesses of several muscles when evaluated separately, and there was also an association of frailty (binary variable) with component 1. It was previously seen that an increase in echogenicity of the quadriceps femoris is positively associated with age and negatively associated with muscle thickness and isometric knee extensor strength (35). In a big cohort of healthy men, the increase of echogenicity of the anterior thigh muscle compartment was related to diminished muscle strength (36). Nevertheless, in our study, no correlation was observed between echogenicity, and frailty. To justify our finding, it is important to point out that the most expressive gain in echogenicity already occurs in middle-aged individuals and may precede the loss of muscle mass (37).

Regarding the surgical outcomes associated with frailty, non-frail patients had fewer surgical complications, as assessed by the Clavien-Dindo Classification scale. There was a positive relationship between longer hospital stay and frailty. Readmissions within one year of follow-up were not correlated with frailty. No statistical correlation between death and frailty was observed. Studies indicate that the aging process varies between individuals depending on their overall functional reserves. This is reflected, for example, in a higher risk of poorer outcomes, given a surgical intervention, including greater chances of mortality (38).

Makary et al. studied patients undergoing elective surgery and demonstrated an increased risk of bad outcomes related to an increasing degree of frailty (39). McAdams-DeMarco et al. also demonstrated that frail patients undergoing kidney transplantation were significantly more likely to be readmitted earlier to the hospital, regardless of age (40). Frailty was directly related to an increase in the number of complications related to colorectal, cardiac and emergency surgery (41, 42).

In our study, considering the same outcomes, now faced with sarcopenia, lower gait speeds were associated with higher proportions of postoperative complications (Clavien-Dindo Classification equal to or above II), longer total hospital stay, and higher death rates. In the sarcopenia (loss of strength criterion), lower handgrip strengths were associated with higher incidence of post-surgical complications: Clavien-Dindo Classification equal to or above II and longer total hospital stay. No association with readmission or death was observed.

The mortality rate up to 1 year after surgery was 4% in our study group and it was especially related to sarcopenia (80% of these patients had severe sarcopenia). Of the 34 patients who underwent readmissions, 43.2% had at least 1 criterion to define sarcopenia or frailty.

## 5. Conclusion

In our study, sarcopenia, in all its domains, was associated with frailty. Unfavorable surgical outcomes were also associated with these two conditions.

Our findings also suggest that screening for sarcopenia and frailty in elderly patients who will undergo elective surgery is relevant, easy-to-perform and helps to access perioperative risk in this population.

We recognize, as limitations of this study, that this is a small sample in which the recruitment occurred in a single private hospital, that may cause restrictions to the generalization of our findings.

The present study is the first, to our knowledge, to assess sarcopenia in all its domains, with frailty, in a presurgical elderly population, with a 1 year follow up. The use of well-defined cutoff parameters, by the latest consensus of the EWGSOP 2019 contributes to create a more universal language for future studies in this area.

## Data availability statement

The raw data supporting the conclusions of this article will be made available by the authors, without undue reservation.

## Ethics statement

The studies involving human participants were reviewed and approved by Hospital Sirio Libanes. The patients/participants provided their written informed consent to participate in this study.

## Author contributions

IS: conception and design of the work and acquisition of data for the work. CT and JS: substantial contributions to the analysis and interpretation of data for the work. CB: revising the article–intellectual content. AA: substantial contributions on acquisition of data for the work. RS: substantial contributions to the conception or design of the work. AB: substantial contributions to the conception or design of the work and accountable for all aspects of the work in ensuring that questions related to the accuracy or integrity of any part of the work are appropriately investigated and resolved. All authors contributed to the article and approved the submitted version.

## Conflict of interest

The authors declare that the research was conducted in the absence of any commercial or financial relationships that could be construed as a potential conflict of interest.

## Publisher’s note

All claims expressed in this article are solely those of the authors and do not necessarily represent those of their affiliated organizations, or those of the publisher, the editors and the reviewers. Any product that may be evaluated in this article, or claim that may be made by its manufacturer, is not guaranteed or endorsed by the publisher.
