# Supplementary material for: Sarcopenia, frailty, and elective surgery outcomes in the elderly: an observational study with 125 patients (the SAFESOE study)
Source: Front Med (Lausanne). 2023 Aug 7;10:1185016. doi: 10.3389/fmed.2023.1185016 (PMC10442161; doi:10.3389/fmed.2023.1185016)

**Appendix 1**. Characteristics of the population studied according to frailty degrees.

| **Categories*** | **Normal**  **(N=77)** | **Pré-Frail**  **(N=33)** | **Frail**  **(N=15)** | **Total**  **(N=125)** | **p-****  **value** |
| --- | --- | --- | --- | --- | --- |
| Gender | | | | | |
| male | 35 (45,5) | 20 (60,6) | 11 (73,3) | 66 (52,8) | 0,082 |
| female | 42 (54,5) | 13 (39,4) | 4 (26,7) | 59 (47,2) |  |
| Age | | | | | |
|  | 68,0 (64,0-74,0) | 72,0 (67,0-78,0) | 78,0 (74,0-83,0) | 71,0 (65,0-77,0) | 0,001 |
| Weight | | | | | |
|  | 73,0 (62,0-84,5) | 74,0 (67,0-84,0) | 69,4 (63,0-75,0) | 73,0 (65,0-84,0) | 0,50 |
| Heigh | | | | | |
|  | 1,7 (1,6-1,7) | 1,7 (1,6-1,7) | 1,7 (1,6-1,7) | 1,7 (1,6-1,7) | 0,67 |
| BMI | | | | | |
|  | 26,6 (23,8-29,7) | 27,1 (23,1-30,8) | 25,8 (22,3-27,5) | 26,6 (23,2-29,8) | 0,37 |
| Bioimpedance | | | | | |
| Tx. Bas. metab. basal | 1487 (1246-1772) | 1528 (1341-1771) | 1374 (1312-1656) | 1471 (1288-1746) | 0,65 |
|  |  |  |  |  |  |
| % fat | 33,8 (28,2-37,9) | 33,4 (26,3-36,2) | 31,9 (26,4-37,7) | 33,3 (27,9-37,7) | 0,60 |
|  |  |  |  |  |  |
| Body water | 49,9 (46,2-55,0) | 50,8 (47,5-55,7) | 52,6 (50,5-59,3) | 50,7 (46,8-55,4) | 0,12 |
| Physical activity | | | | | |
| No | 37 (48,1) | 21 (63,6) | 8 (53,3) | 66 (52,8) | 0,27 |
| Parcial | 22 (28,5) | 9 (27,3) | 6 (40,0) | 37 (29,6) |  |
| Yes | 18 (23,4) | 3 (9,1) | 1 (6,7) | 22 (17,6) |  |

| Number of different Medicines | | | | | |
| --- | --- | --- | --- | --- | --- |
|  | 3,0 (2,0-5,0) | 6,0 (5,0-8,0) | 6,0 (4,0-9,0) | 4,0 (2,0-6,0) | <0,001 |
| Comorbidities | | | | | |
|  | 3,0 (2,0-4,0) | 4,0 (3,0-5,0) | 5,0 (4,0-6,0) | 3,0 (2,0-4,0) | <0,001 |
| ASA | | | | | |
| 1 | 11 (14,3) | 1 (3,0) | 0 (0,0) | 12 (9,6) | <0,001 |
| 2 | 59 (76,6) | 21 (63,7) | 5 (33,3) | 85 (68,0) |  |
| 3 | 7 (9,1) | 11 (33,3) | 10 (66,7) | 28 (22,4) |  |

* Data are presented as median (IIR) for continuous measures and n (%) for categorical measures.

** Kruskal-Wallis t test for continuous measures and Fisher's Exact or Chi-square tests for categorical measures. Significant results at the 5% significance level are highlighted in red.

**Appendix 2**. Ultrasound cross-sectional area of ​​eight muscle groups

gc: gastrocnemius medialis


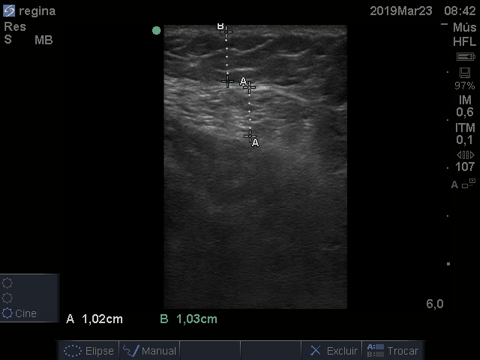


eo: external oblique/io: internal oblique/ta: transversus abdominais


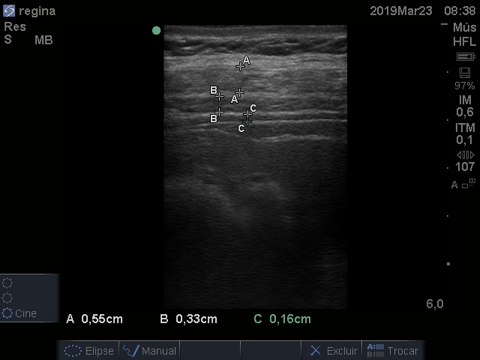


ra: rectus abdominis


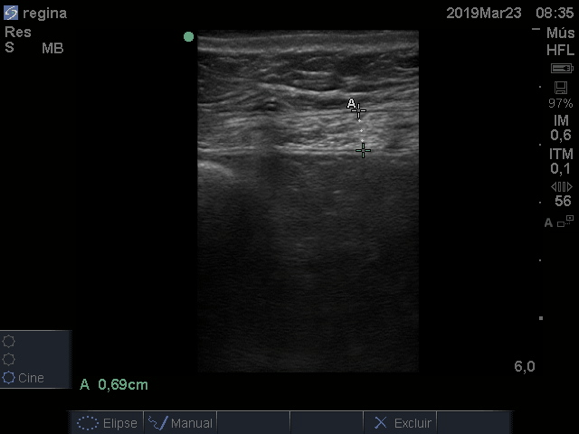


rf: rectus femoris/ vi: vastus intermediate: Quadriceps


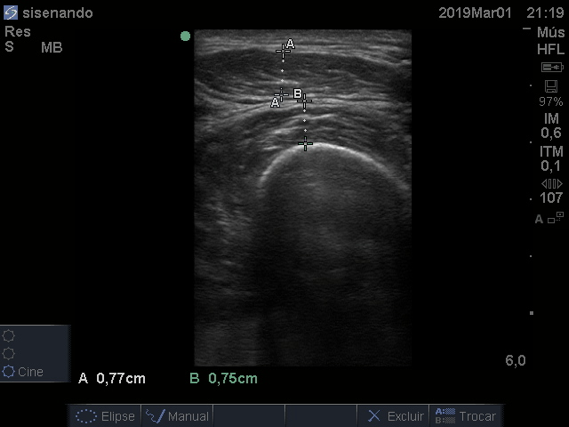


bb: biceps brachii and brachialis


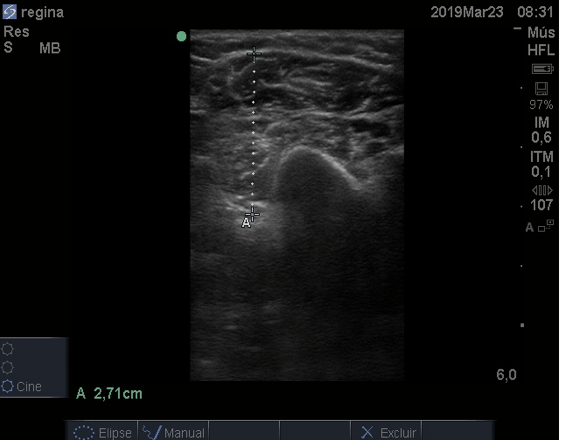


**Appendix 3.** The Clavien-Dindo Classification


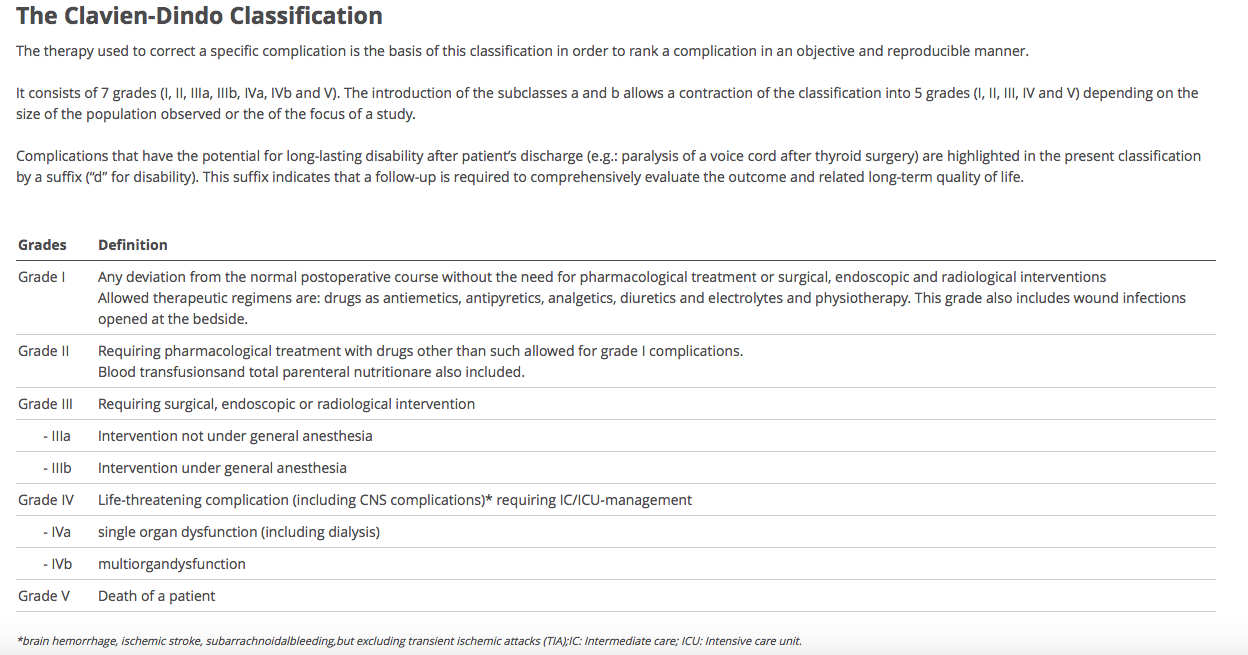

Supplement: Supplementary file 1 [file Data_Sheet_1.docx]
